# Supplementary material for: The Biodistribution of Replication-Defective Simian Adenovirus 1 Vector in a Mouse Model
Source: Viruses. 2024 Mar 31;16(4):550. doi: 10.3390/v16040550 (PMC11054548; doi:10.3390/v16040550)
Supplement: Supplementary file 1 [file viruses-16-00550-s001.zip › viruses-2906052-supplementary.pdf]

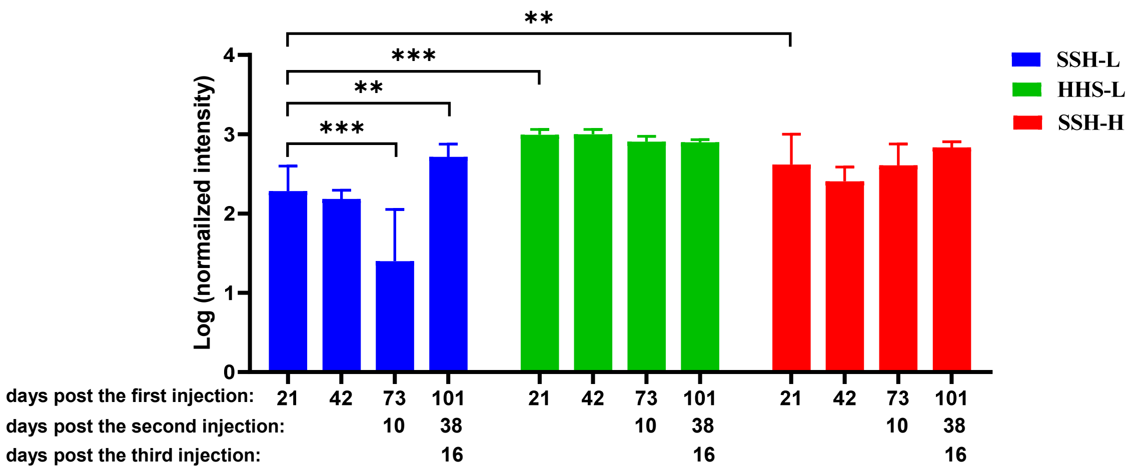


**Figure S1.** Immunofluorescence assay to detect serum antibodies against reporter GFP protein. After the first injection, HHS-L group generated highest level of anti-GFP antibodies, and the level was maintained throughout the experimental duration. In SSH-L group, sequential inoculation of HAdV5-GFluc significantly increased the level of serum antibody against GFP. After the third injection, there was no significant difference in serum antibody level between groups. ** $p<0.01$, ***$P<0.001$. The experimental procedure was described below.

A plasmid carrying CMV promoter-controlled GFP gene (pLEGFP-C1, CLONTECH) was used to transfect 293 cells. The next day, the cells were detached by trypsin treatment, dispersed into single cells, seeded in 96-well plates and cultivated for another 24 hours. The percentage of GFP+ cells is approximately 42%. Cells was fixed in 100 µl 4% polyformaldehyde in PBS per well at room temperature for 20 miniutes, washed with PBS for 3 times, penetrated by 0.2% Triton X-100 at room temperature for 10 minutes, washed with PBS for 3 times, blocked with PBS plus 1% BSA at room temperature for 30 minutes, incubated with 1:50 diluted mouse sera at room temperature for 2 hours (50 µl per well, in duplicate), washed with PBST for 3 times (250 µl per well), incubated with 1:100 diluted TRITC-conjugated goat anti-mouse IgG (Cat. ZF-0313, ZSGB-BIO, Beijing, China) at room temperature for 1 hour and washed with PBST for 3 times. Cells with red fluorescence signal were photographed by using a high-throughput image analyzer (Countstar Castor, Alit Biotech, Shanghai, China). The fluorescence intensity per well was calculated by using Fiji image processing package (http://fiji.sc/). Briefly, open image> image|color| split channels> using the red channel image > oval tool to select the whole well > edit | clear outside > image | adjust | threshold > analyze | set measurements (select intensity density) > analyze | measure (the steps were recorded as a Macro programming and used to analyzed images of other wells). The fluorescence intensity was log-transformed and normalized by subtracting the mean value of negative sera from PBS mock-infected mice.
